# Analysis of physical characteristics of Tumor Treating Fields for human glioblastoma

**DOI:** 10.1002/cam4.1095

**Published:** 2017-05-23

**Authors:** Edwin Lok, Pyay San, Van Hua, Melissa Phung, Eric T. Wong

**Affiliations:** ^1^ Brain Tumor Center & Neuro‐Oncology UnitBeth Israel Deaconess Medical CenterHarvard Medical SchoolBostonMassachusetts; ^2^ Department of Physics and Applied PhysicsUniversity of Massachusetts in LowellLowellMassachusetts

**Keywords:** Conductivity, disposition, glioblastoma, malignant gliomas, multiphysics, tumor geometry, Tumor Treating Fields

## Abstract

Tumor Treating Fields (TTFields) therapy is an approved treatment that has known clinical efficacy against recurrent and newly diagnosed glioblastoma. However, the distribution of the electric fields and the corresponding pattern of energy deposition in the brain are poorly understood. To evaluate the physical parameters that may influence TTFields, postacquisition MP‐RAGE, T1 and T2 MRI sequences from a responder with a right parietal glioblastoma were anatomically segmented and then solved using finite‐element method to determine the distribution of the electric fields and rate of energy deposition at the gross tumor volume (GTV) and other intracranial structures. Electric field–volume histograms (EVH) and specific absorption rate–volume histograms (SARVH) were constructed to numerically evaluate the relative and/or absolute magnitude volumetric differences between models. The electric field parameters E_AUC_, V_E__150_, E_95%_, E_50%_, and E_20%_, as well as the SAR parameters SAR_AUC_, V_SAR__7.5_, SAR_95%_, SAR_50%_, and SAR_20%_, facilitated comparisons between models derived from various conditions. Specifically, TTFields at the GTV were influenced by the dielectric characteristics of the adjacent tissues as well as the GTV itself, particularly the presence or absence of a necrotic core. The thickness of the cerebrospinal fluid on the convexity of the brain and the geometry of the tumor were also relevant factors. Finally, the position of the arrays also influenced the electric field distribution and rate of energy deposition in the GTV. Using EVH and SARVH, a personalized approach for TTFields treatment can be developed when various patient‐related and tumor‐related factors are incorporated into the planning procedure.

## Introduction

Tumor treating fields (TTFields) are alternating electric fields, tuned to a frequency between 100 and 300 kHz, that have antimitotic properties against rapidly dividing cancer cells. When properly applied, these fields disrupt macromolecular protein structures thought to possess large dipole moments, such as Tubulin and Septin, which are critical for proper cytokinesis 1, 2, 3. In past randomized clinical trials, TTFields was shown to have comparable efficacy against recurrent glioblastoma when compared to chemotherapies and, when added to maintenance temozolomide, it had a superior benefit in newly diagnosed glioblastoma patients when compared to maintenance temozolomide alone 4, 5. Although the clinical efficacy against glioblastoma is apparent, the intracranial distribution of the electric fields and to what extent the dose of these fields retard tumor growth remain largely unknown.

TTFields are applied to the shaved scalp via two pairs of orthogonally positioned transducer arrays. Clinical placement of the arrays is determined by the proprietary NovoTAL^TM^ software that generates an array layout diagram 6. Each array has nine ceramic disks acting like disk sources for the electric fields. However, unlike high‐energy ionizing radiation that can penetrate intracranial structure in a straight beam path, the intensity and directionality of electric fields are heavily influenced by the local dielectric properties of various structures in the head, particularly the electric conductivity and the relative permittivity of brain tissue 7. As the electric conductivity and relative permittivity of tissues vary, the absorption and attenuation of TTFields will change and thus the distribution of these fields will be distorted as they permeate throughout the brain. In addition, there is currently no standardized electric conductivity and relative permittivity values for glioblastoma. As each individual tumor will vary in size, geometry, location within the brain, cellular composition, and presence or absence of necrosis, all of which may influence the electric field and energy absorption distribution within the glioblastoma. Therefore, to properly evaluate how tissue dielectric properties and physical characteristics of the various tissues influence the applied TTFields in the glioblastoma patient, a computer simulated model, currently best solved by the finite‐element method, is needed. This model takes into account the normal brain structures, the gross tumor volume (GTV), the presence or absence of a necrotic core within the tumor, and local tissue electric conductivity and relative permittivity values. Here, we performed detailed finite‐element modeling of a patient who responded to TTFields treatment. We found that there is heterogeneity in the electric field intensity and the rate of energy absorbed at the GTV depending on the placement of the transducer arrays, the presence or absence of a necrotic core within the glioblastoma, the thickness of the cerebrospinal fluid on the convexity of the brain and tumor geometry. The intensity of the electric fields and the specific absorption rate (SAR) were also represented graphically by the electric field‐volume histogram (EVH) and the specific absorption rate–volume histogram (SARVH), respectively.

## Materials and Methods

Critical neuroanatomical structures such as the scalp, skull, dura, cerebrospinal fluid, white matter, gray matter, brainstem, cerebellum, orbits, and bilateral ventricles were segmented based on the postacquisition MP‐RAGE image dataset from a responder with a glioblastoma in the right parietal brain. The segmentation of these masks was performed using ScanIP (Simpleware LTD., UK) where grey‐scale thresholding methods were applied to initially segmented tissues, followed by manual correction on these masks. Additionally, T1 and T2 MRI sequences were also imported into ScanIP, and then coregistered with the segmented masks for delineation of the GTV and necrotic core; the remaining unsegmented tissue was labeled as unspecified tissue and given material properties that of muscle. Structures such as orbits, brainstem, and cerebellum were segmented and their associated physical properties were included mainly for spatial reference in the model. The GTV and necrotic core were both manually segmented by the treating physician based on the visible enhancement shown on the coregistered postgadolinium T1‐ and T2‐weighted image datasets. Transducer arrays were manually placed on the surface of the scalp in the model, approximating as closely as possible to the standard display as shown in the United States Food and Drug Administration's communication on the NovoTTF‐100A system 8. Upon completion of segmentation, a three‐dimensional finite‐element mesh was generated within ScanIP. This mesh was then imported into COMSOL Multiphysics (COMSOL, Burlington, MA), where material properties, boundary conditions, and appropriate physics parameters were assigned and applied.

For the purpose of demonstrating the sensitivity of varying physical properties, such as electric conductivity and relative permittivity, tumor geometry, array disposition, the introduction or elimination of certain intracranial structures, and the expansion or contraction of cerebrospinal fluid volume, only isotropic electric conductivity values were used in order to simplify the modeling process. Data postprocessing of the solved models was performed using Microsoft Excel, including generation of volume histograms EVH and SARVH. Area under the curve was computed using Simpson's Rule integration in MATLAB 2016 for electric fields (E_AUC_) and SAR (SAR_AUC_). The EVH was used in the comparison of electric field strength between different models and was referenced to (1) the percentage volume of a particular structure receiving at least 150 V/m (V_E150_), (2) the magnitude of electric field strength encompassing 95% of a particular structure's volume (E_95%_), (3) the magnitude of electric field strength encompassing 50% of a particular structure's volume (E_50%_), and (4) the magnitude of electric field strength encompassing 20% of a particular structure's volume (E_20%_). Similarly, the comparison of the rate of energy absorbed in different SARVH models was referenced to (1) the percentage volume of a particular structure receiving at least 7.5 W/kg (V_SAR7.5_), (2) the magnitude of SAR encompassing 95% of a particular structure's volume (SAR_95%_), (3) the magnitude of SAR encompassing 50% of a particular structure's volume (SAR_50%_), and (4) the magnitude of SAR encompassing 20% of a particular structure's volume (SAR_20%_).

## Results

### Electric field distribution of a responder treated with TTFields

**Figure 1:**
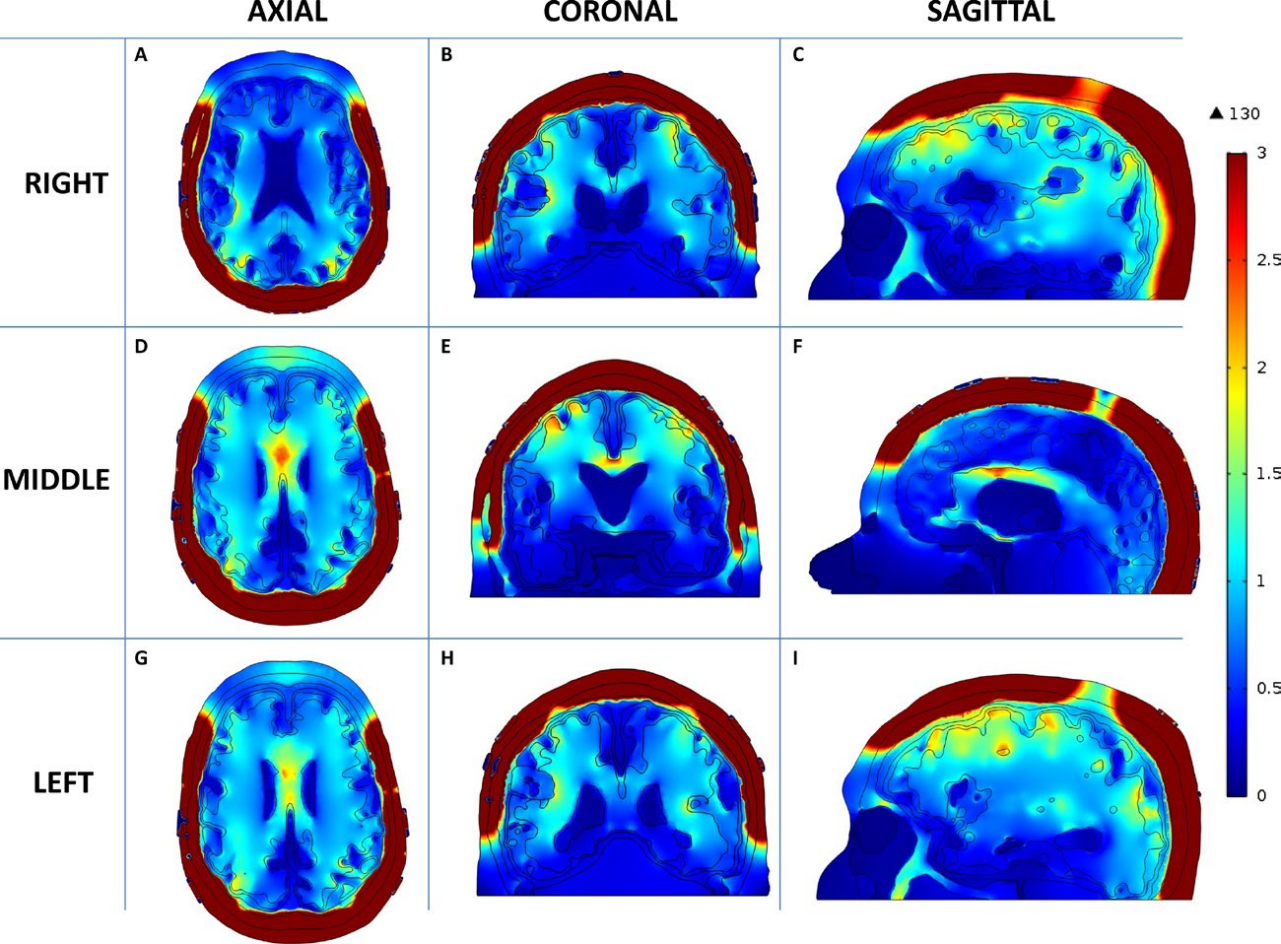
Distribution of TTFields within a patient brain. The electric field intensity is higher in the supratentorial than the infratentorial brain. Within the supratentorial brain, various parts of the sulci, the body of the corpus callosum, and the medial surface of the GTV appear to have the highest electric field intensity. Axial images from inferior to superior slices are shown in A, D and G. Coronal images from anterior to posterior slices are shown in B, E and H. Sagittal images from right to left slices are shown in C, F and I. TTFields, Tumor Treating Fields; GTV gross tumor volume.

A retrospective analysis of one glioblastoma patient, who responded to TTFields treatment, was performed by modeling the intracranial electric field distribution using finite‐element analysis. The responder had a glioblastoma in the right parietal lobe extending toward the bilateral ventricles with roughly 1.5 cm between the GTV and the lateral border of the right lateral ventricle. As expected, the highest electric field intensity was seen within the sulci on the surface of the brain and it is associated with a high‐to‐low gradient from the surface to the deeper regions (Fig. 1). In particular, the lowest intensity was seen in the inferior portion of the frontal (Fig. 1C and I) and the temporal (Figs. 1B and H) lobes. Furthermore, the body of the corpus callosum had high electric field intensity, particularly in the regions between (Fig. 1D) and above the lateral ventricles (Fig. 1E and F). This is likely due to the relatively higher electric conductivity of the cerebrospinal fluid located next to either side of the lesser conductive white matter, which probably created a higher capacitive reactance similar to a capacitor, helped to retain a higher electric field intensity within the white matter. Lastly, the medial portion of GTV in the right parietal lobe also possessed a higher electric field intensity. This could be a result of a relatively higher electric conductivity of the necrotic core within the GTV on one side and the cerebrospinal fluid within the right lateral ventricle on the other side, both of which also likely contributed to a higher reactive capacitance within the right parietal tissue.

**Figure 2:**
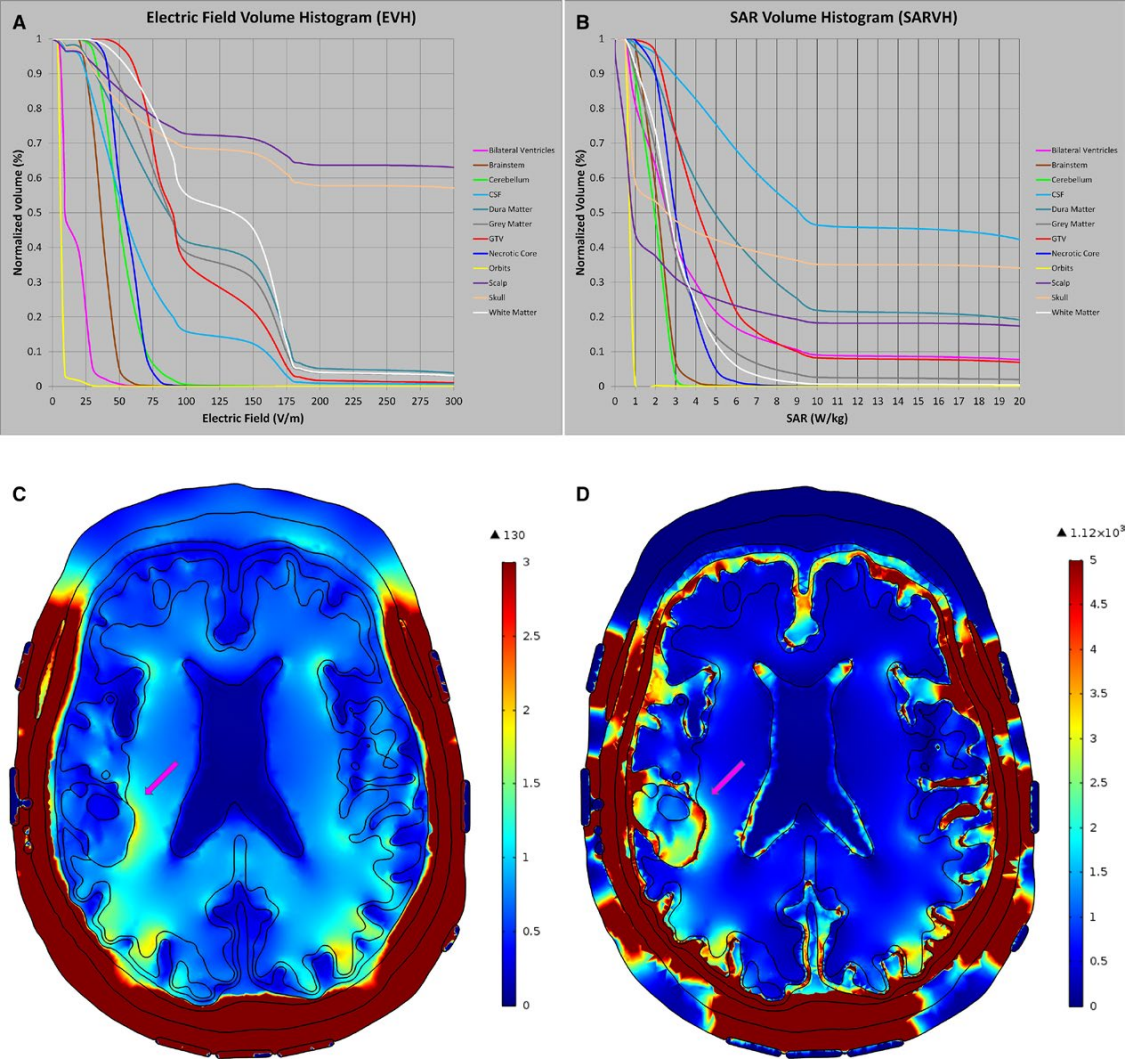
Volume histograms EVH and SARVH. The EVH (A), SARVH (B), electric field map (C), and SAR map (D) were generated using the transducer array placement as outlined in Figure 3E. The highest E_AUC_ was found at the scalp and skull, whereas the lowest was detected at the orbits, bilateral ventricles, and brainstem. The highest SAR_AUC_ was found at the skull, GTV, and the layer of cerebrospinal fluid between cortex and dura, whereas the lowest was found in the orbits, cerebellum, and the orbits. EVH, electric field–volume histogram; SARVH, specific absorption rate–volume histogram; SAR, specific absorption rate.

**Figure 3:**
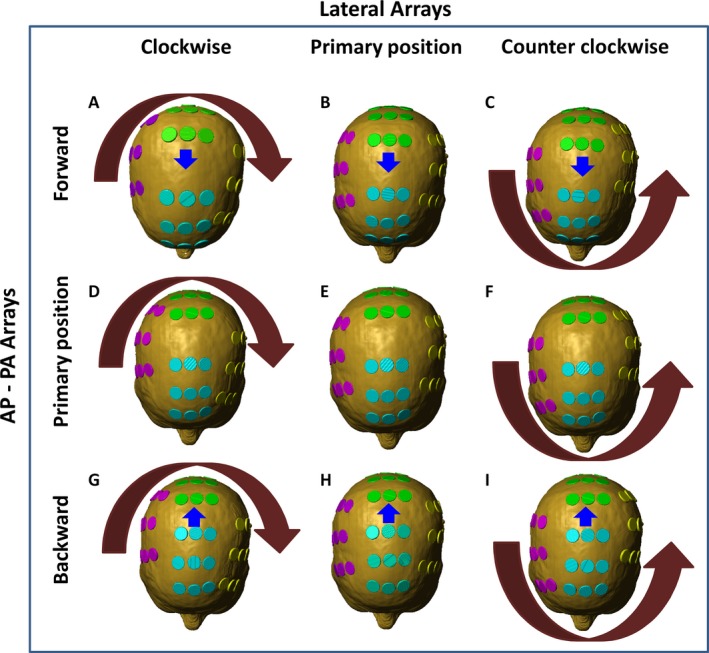
Array displacement for disposition analysis. The original array placement position was determined by the NovoTAL^TM^ software that generated an array placement diagram (E). The disks in each array were then rotated in aggregate by 2‐cm deviation from the primary position in a clockwise fashion (A, D, and G) and counterclockwise fashion (C, F, and I). In addition, the posterior arrays were moved in aggregate by 2‐cm deviation forward (A, B, and C) and backward (G, H, and I).

To investigate the strength of electric field and the rate of energy deposited into the GTV and various intracranial structures, EVH and SARVH were generated for the comparison between models that use the primary position for transducer array placement as outlined in Figure 3E and incorporate the isotropic electric conductivity and relative permittivity values listed in Table 1 9, 10. As expected, the highest E_AUC_ was found at the scalp and skull, whereas the lowest was located at the orbits, bilateral ventricles, and brainstem (Fig. 2A and C). In the GTV, 95% of the volume had an electric field intensity of >50 V/m, whereas 50% had >80 V/m and 20% has >150 V/m (Fig. 2A). Because at least 20% of the GTV volume had a minimum of 150 V/m of electric field coverage (Table 2), the V_E150_ volume and E_50%_ magnitude were used as means of comparing different modeling outcomes in subsequent analyses. Similarly, the highest SAR_AUC_ was found at the skull, GTV, and the layer of cerebrospinal fluid between cortex and dura, whereas the lowest were located in the orbits, cerebellum, and brainstem (Fig. 2B and D). Because at least 15% of the GTV volume had a SAR of at least 7.5 W/kg (Table 2), the V_SAR7.5_ volume and SAR_50%_ magnitude were used as means of comparing different modeling outcomes in subsequent analyses.

**Table 1:** Physical parameters required as inputs for computer modeling

Tissue structure	Volume (cc)	Electric conductivity *σ* (S/m)	Relative permittivity ε_r_
Gross tumor volume (GTV)	5.813874	2.50E‐01	1.00E+04
Necrotic core	2.421458	1.00E+02	1.00E+00
Scalp	524.5453	1.05E‐03	1.10E+03
Skull	463.5451	2.11E‐02	2.04E+02
Dura	216.8171	5.02E‐01	2.90E+02
Cerebrospinal fluid	238.8805	2.00E+00	1.09E+02
White matter	593.1396	8.68E‐02	1.29E+03
Gray matter	261.5665	1.41E‐01	2.01E+03
Bilateral ventricle	51.38429	2.00E+00	1.09E+02
Brainstem	28.7721	1.61E‐01	2.30E+03
Orbits	12.89734	1.50E+00	9.66E+01
Cerebellum	44.55224	1.61E‐01	2.30E+03
Unspecified tissue/muscle	133.3064	3.84E‐01	6.38E+03
Electrodes	N/A	1.00E‐05	1.10E+04
Titanium wires	N/A	1.28E+06	5.00E+01

The volume, electric conductivity and relative permittivity values for GTV, necrotic core, scalp, skull, dura, cerebrospinal fluid, white matter, gray matter, bilateral ventricles, brainstem, orbits, cerebellum, unspecified tissue/muscle, electrodes, and titanium wires that were used in the analysis.

**Table 2:** The values for electric field and rate of energy deposition parameters at the GTV and other structures in the head as shown in EVH and SARVH from Figure 2

Tissue structure	E_AUC_	V_E150 (%)_	E_95%_	E_50%_	E_20%_	SAR_AUC_	V_SAR7.5 (%)_	SAR_95%_	SAR_50%_	SAR_20%_
Bilateral ventricle	14.7	0.0	5.7	9.8	25.2	4.0	13.1	0.7	2.6	5.3
Brainstem	35.7	0.0	23.6	36.3	44.0	1.2	0.0	1.2	2.1	2.6
Cerebellum	50.4	0.1	32.0	49.0	62.6	1.0	0.0	0.8	2	2.5
Cerebrospinal fluid	67.3	12.3	21.0	53.5	90.2	19.0	58.6	2.2	9.2	30
Dura	108.3	35.7	25.4	86.2	169.8	8.2	32.3	1.3	4.9	17.1
Gray matter	108.1	31.0	37.7	87.2	164.7	2.5	5.6	0.8	2.6	4.3
Gross tumor volume (GTV)	104.0	21.7	57.0	89.7	153.5	5.9	13.9	2.1	4.1	6.2
Necrotic core	54.1	0.0	38.8	53.8	65.2	3.5	5.6	2.3	4.1	5.8
Orbits	5.7	0.0	4.2	6.2	7.8	0.0	0.0	0.5	0.8	0.9
Scalp	596.0	71.2	24.8	>1000	>1000	8.7	21.0	0.1	0.9	8.3
Skull	537.8	67.1	24.6	437.2	>1000	31.8	38.1	0.6	2.5	42.8
White matter	126.9	45.3	48.7	137.0	169.8	2.2	2.4	0.9	2.7	4.3

GTV, gross tumor volume; EVH, electric field–volume histogram; SARVH, specific absorption rate–volume histogram; E_AUC_, electric field area under the curve; V_E150_, volume covered with electric field intensity of 150 volts per meter; E_95%_, the electric field intensity encompassing 95% of volume; E_50%_, the electric field intensity encompassing 50% of volume; E_20%_, the electric field intensity encompassing 50% of volume; SAR, specific absorption rate; SAR_AUC_, SAR area under the curve; V_SAR7.5_, volume covered with specific absorption rate of 7.5 watts per kilogram; SAR_95%_, the magnitude of specific absorption rate encompassing 95% of volume; SAR_50%_, the magnitude of specific absorption rate encompassing 50% of volume; SAR_20%_, the magnitude of specific absorption rate encompassing 20% of volume.

### Disposition analysis of transducer array placement

**Figure 4:**
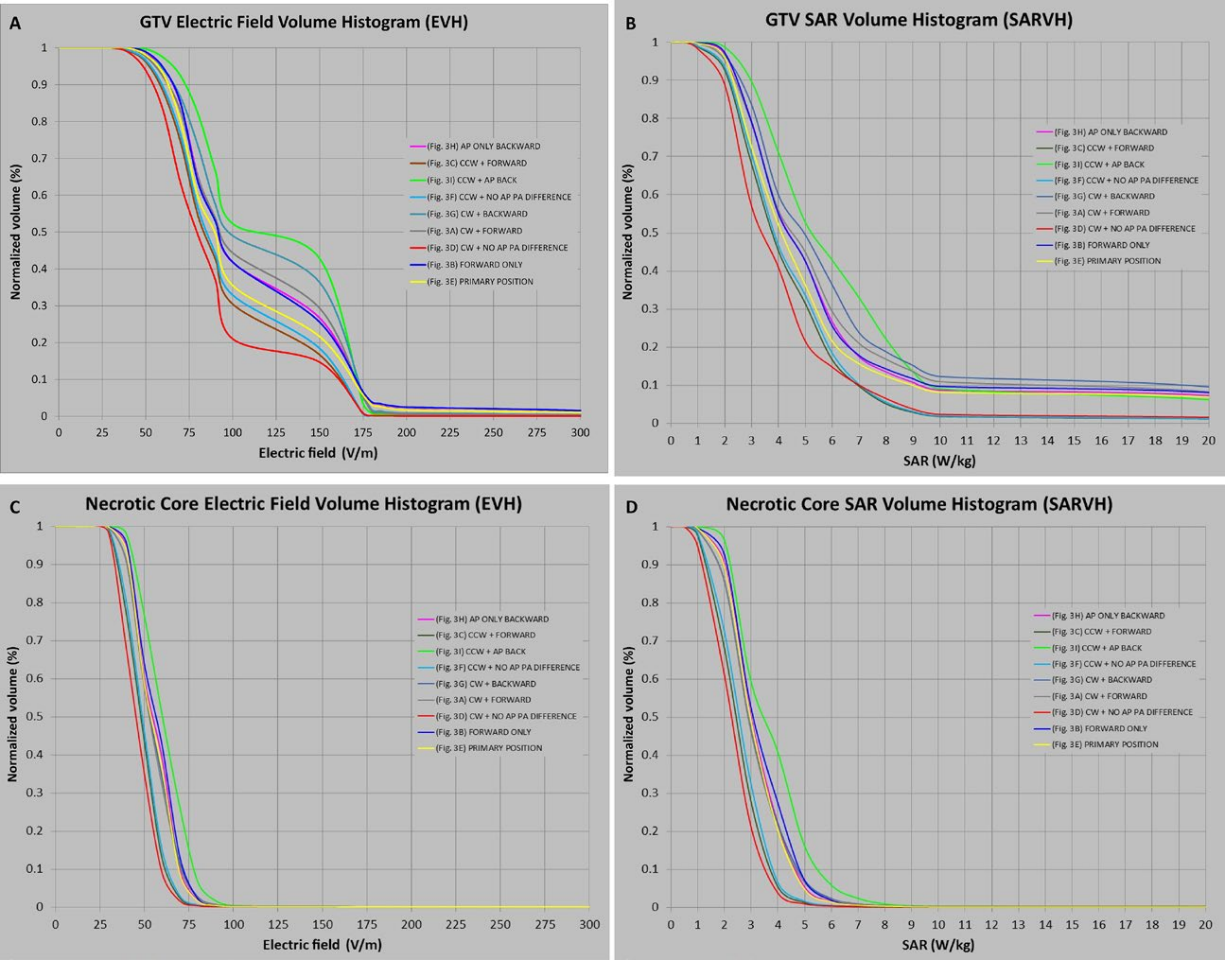
EVH and SARVH generated from array displacement analysis. Electric field coverage of the GTV was highly variable between 100 and 150 V/m, ranging from only 20% volume having 100 V/m with clockwise rotation of the lateral arrays and no displacement of the anterior–posterior arrays (red curve), to >40% volume having at least 150 V/m when the lateral arrays were rotated in a counterclockwise fashion and the anterior array was moved backward (green curve) (A). However, the variability in the magnitude of SAR encompassing 50% of the GTV, as represented by SAR_50%_, was low and it ranged between 3 and 6 W/kg (B). For the necrotic core of the tumor, the electric field (C) and SAR magnitude (D) variability at the GTV were also smaller. Each color‐coded curve corresponds to specific array placement position as shown in Figure 3A: CW+Forward, Figure 3B: Forward Only, Figure 3C: CCW+Forward, Figure 3D: CW+No AP PA Difference, Figure 3E: Primary Position, Figure 3F: CCW+No AP PA Difference, Figure 3G: CW+Backward, Figure 3H: AP Only Backward, and Figure 3I: CCW+AP Backward. AP, anteroposterior; PA, posteroanterior; CW, clockwise; CCW, counterclockwise; EVH, electric field–volume histogram; SARVH, specific absorption rate–volume histogram; GTV, gross tumor volume; V/m, volt per meter; W/kg, watt per kilogram; SAR, specific absorption rate; SAR_50%_, the magnitude of SAR encompassing 50% of volume.

As the placement of transducer arrays can be shifted during each array exchange on treatment, we investigated whether or not the disposition of the arrays can alter electric field coverage and rate of energy deposition in the GTV. The effects of shifting array positions at different locations on the scalp were modeled according to an aggregate 2‐cm deviation from the primary position; each of the nine disks in each array were manually drawn to scale and placed in full contact with the surface of the scalp in the configuration of the primary position as shown in Figure 3E. The lateral arrays were then shifted in clockwise or counterclockwise configurations, whereas the anterior–posterior arrays were shifted in the forward or backward positions, resulting in eight additional configurations (Fig. 3, except for Fig. 3E [primary position]). The posterior array in all cases was not moved inferiorly due to the fact that the patient's image dataset was truncated at the occiput, and thus had insufficient occipital anatomy to shift the posterior array inferiorly. Still, there was high variance in electric field coverage of the GTV between 100 and 150 V/m, ranging from only 20% volume having 100 V/m with clockwise rotation of the lateral arrays and no displacement of the anterior–posterior arrays (Fig. 4A, red curve and Table 3) to >40% volume having at least 150 V/m when the lateral arrays were rotated in a counterclockwise fashion and the anterior array was moved backward (Fig. 4A, green curve). However, the variability in SAR, as represented by the magnitude of SAR_50%_, was low and it ranged between 3 and 6 W/kg (Fig. 4B). For the necrotic core of the tumor, there was also a similar but smaller variance in the electric field coverage (Fig. 4C) and its corresponding SAR magnitude (Fig. 4D).

**Table 3:** Disposition analysis of transducer array placement on EVH and SARVH

Structure	Array position	EVH				SARVH			
		V_E150 (%)_	E_95%_	E_50%_	E_20%_	V_SAR7.5 (%)_	SAR_95%_	SAR_50%_	SAR_20%_
GTV	AP Backward Only	26.71	59.2	92.5	159.2	15.20	2.2	4.4	6.7
	CCW + Forward	16.70	52.2	83.5	139.5	7.10	1.8	3.7	5.7
	CCW AP backward	42.84	65.7	107.0	164.5	27.49	2.5	5.2	8.2
	CCW No AP PA difference	18.44	53.1	85.0	146.0	7.53	1.8	3.8	5.9
	CW backward	36.35	58.3	98.2	164.4	20.92	2.2	5.0	7.7
	CW forward	29.34	55.6	93.5	161.1	8.09	1.5	3.4	5.2
	CW No AP PA Difference	14.70	48.3	79.4	104.0	8.09	1.5	3.4	5.2
	Forward only	25.56	59.4	92.5	158.4	15.78	2.2	4.4	6.6
	No Shift	21.66	57.0	89.7	153.5	13.88	2.1	4.1	6.2
Necrotic Core	AP backward only	0.04	39.4	54.7	65.8	0.26	1.7	3.0	4.1
	CCW + forward	0.01	33.4	48.1	56.9	0.10	1.2	2.4	3.3
	CCW AP Backward	0.11	42.6	60.2	72.7	1.41	2.1	3.5	4.8
	CCW No AP PA Difference	0.01	33.9	48.9	57.8	0.12	1.3	2.6	3.4
	CW backward	0.12	36.7	53.3	65.6	0.60	1.5	2.9	4.1
	CW forward	0.10	36.7	52.8	64.9	0.58	1.5	2.9	4.0
	CW No AP PA difference	0.02	32.2	45.3	54.9	0.10	1.0	2.3	3.0
	Forward only	0.05	40.4	56.5	67.1	0.33	1.8	3.1	4.3
	No shift	0.05	38.8	53.8	65.2	0.35	1.6	3.0	4.0

GTV, gross tumor volume; EVH, electric field–volume histogram; SARVH, specific absorption rate–volume histogram; V_E150_, volume covered with electric field intensity of 150 volts per meter; E_95%_, the electric field intensity encompassing 95% of volume; E_50%_, the electric field intensity encompassing 50% of volume; E_20%_, the electric field intensity encompassing 50% of volume; SAR, specific absorption rate; V_SAR7.5_, volume covered with specific absorption rate of 7.5 watts per kilogram; SAR_95%_, the magnitude of specific absorption rate encompassing 95% of volume; SAR_50%_, the magnitude of specific absorption rate encompassing 50% of volume; SAR_20%_, the magnitude of specific absorption rate encompassing 20% of volume. AP, anteroposterior; PA, posteroanterior; CW, clockwise; CCW, counterclockwise.

### Sensitivity analysis of the conductivity of the GTV and the necrotic core

**Figure 5:**
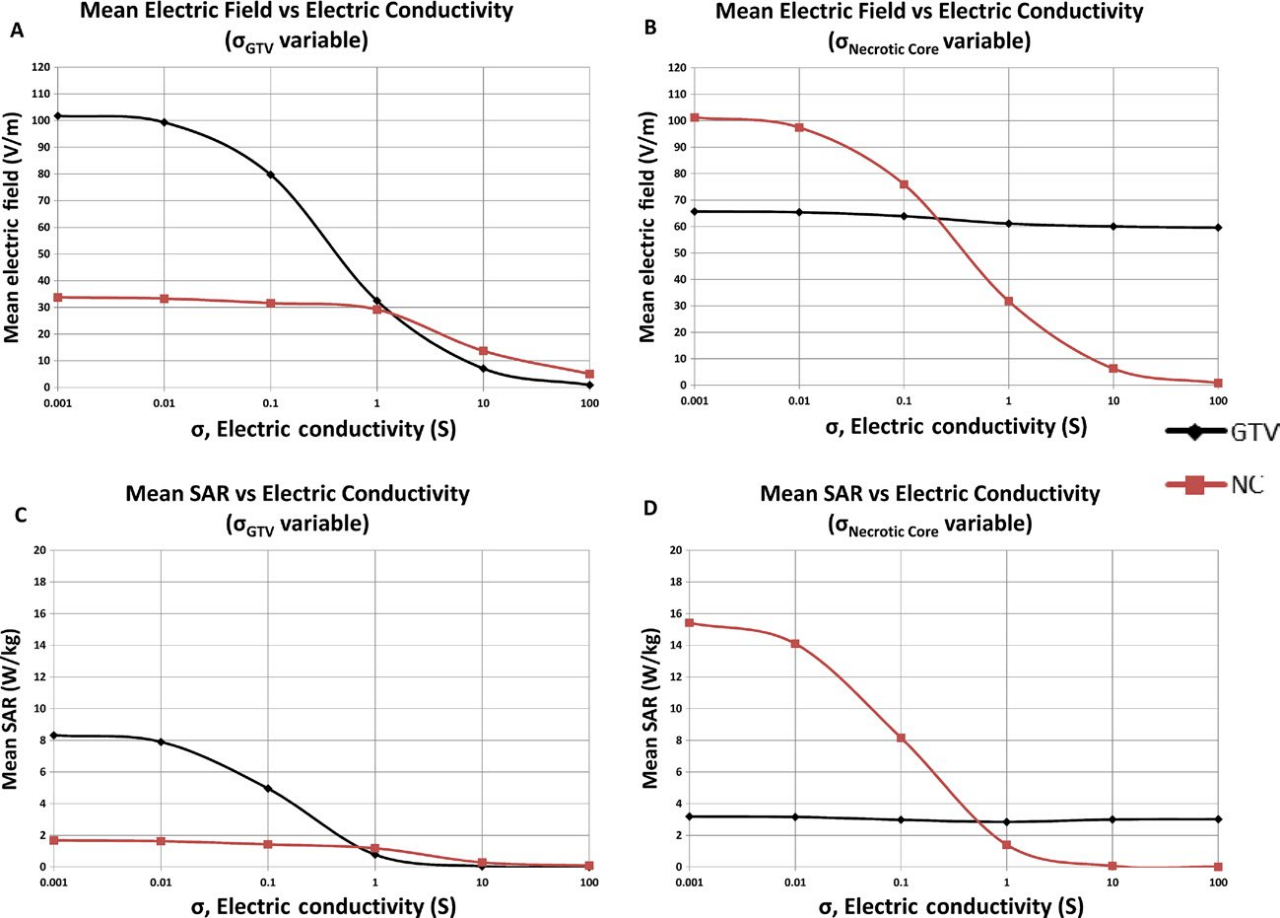
Sensitivity analysis of electric conductivity of GTV and necrotic core. When the electric conductivity of the necrotic core was held constant, the mean electric field strength within the necrotic core rose 600% from 5 to 30 V/m when the conductivity of the GTV decreased from 100 to 1 S/m, but further increase was markedly attenuated when the conductivity was lowered from 1 to 0.001 S/m (A). The mean SAR increased from 0 to 1.8 W/kg when the conductivity of the GTV was decreased from 100 to 1 S/m, but further increase was negligible when the conductivity was lowered from 1 to 0.001 S/m (C). When the electric conductivity of the GTV was held constant and the electric conductivity of the necrotic core was varied from 100 to 1 S/m, there was negligible change in the mean electric field strength in the GTV; but there was up to a 10% increase in mean electric field strength of the GTV when the electric conductivity of the necrotic core was lowered from 1 to 0.001 S/m (B). However, the change in mean SAR of the GTV was insignificant when the necrotic core conductivity was varied from 100 to 0.001 S/m (D). GTV, gross tumor volume; V/m, volt per meter; S/m, Siemens per meter; W/kg, watt per kilogram; SAR, specific absorption rate.

The strength of the electric field penetrating the GTV most likely depends on its dielectric properties, specifically the electrical conductivity and relative permittivity. Our previous modeling found that the electric field intensity at the GTV is more sensitive to changes in its electric conductivity than its relative permittivity characteristics 7. However, glioblastoma frequently has a necrotic component that contains liquefied cellular products or exudates from adjacent highly permeable vasculature. The fluid component of this necrotic core can potentially influence the electric field strength within the GTV. To investigate the relationship between the field coverage at both GTV and necrotic core, the electric conductivity of the GTV was altered from 100 to 0.001 S/m, whereas the electric conductivity of the necrotic core was kept constant. The mean electric field strength within the necrotic core rose 600% from 5 to 30 V/m when the electric conductivity of the GTV decreased from 100 to 1 S/m, but further increase was markedly attenuated when the electric conductivity was lowered from 1 to 0.001 S/m (Fig. 5A). Similarly, the mean SAR increased from 0 to 1.8 W/kg when the electric conductivity of the GTV decreased from 100 to 1 S/m, but further increase was negligible when the electric conductivity was lowered from 1 to 0.001 S/m (Fig. 5C). In contrast, when the electric conductivity of the necrotic core was varied from 100 to 1 S/m, there was negligible change in the mean electric field strength in the GTV; however, there was up to a 10% increase in mean electric field strength of the GTV when the electric conductivity of the necrotic core was lowered from 1 to 0.001 S/m (Fig. 5B). But the change in mean SAR of the GTV was insignificant when the electric conductivity of the necrotic core was varied from 100 to 0.001 S/m (Fig. 5D).

**Figure 6:**
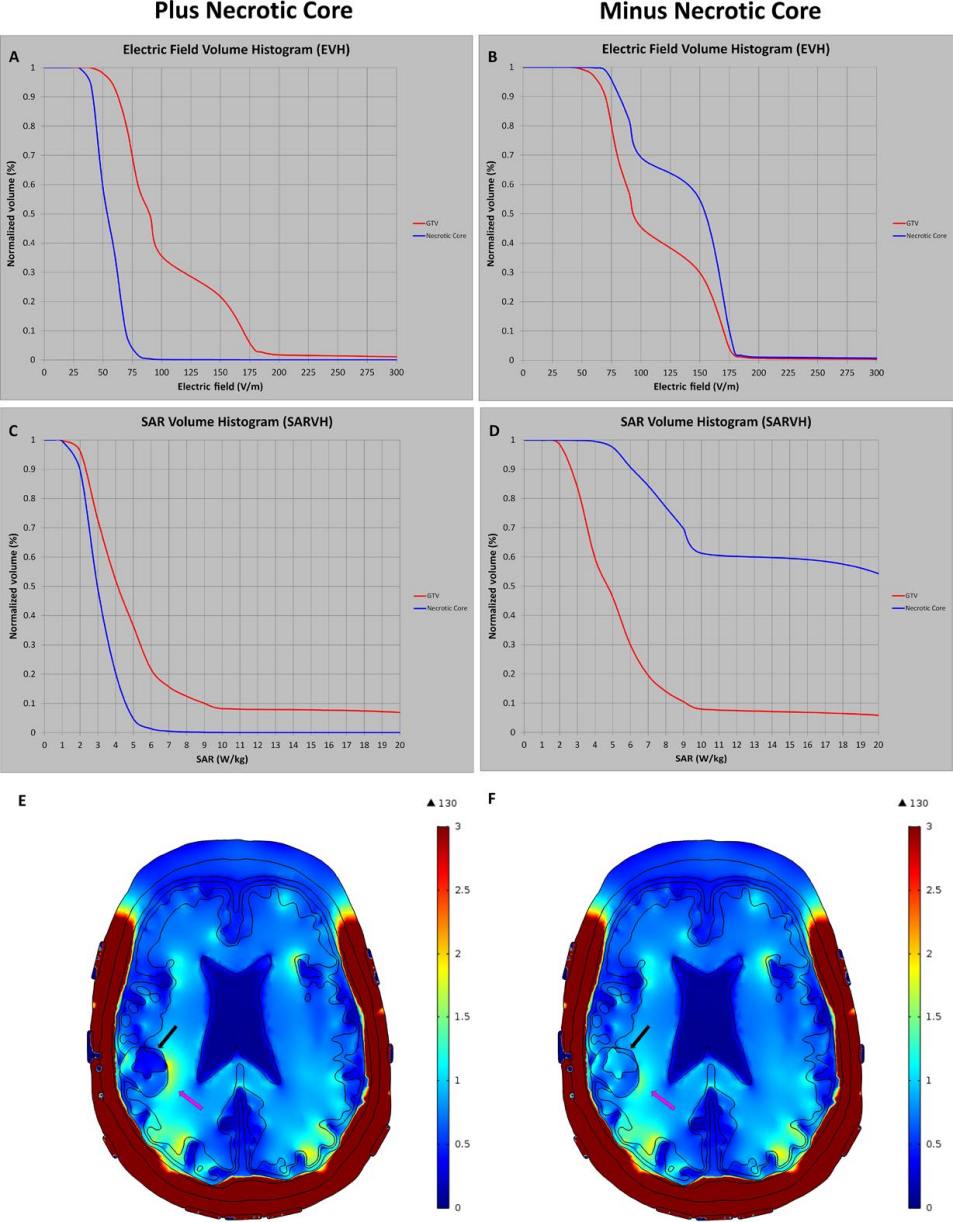
Sensitivity analysis for electric field strength and rate of energy deposition with or without the necrotic core. EVH (A) and SARVH (C) were modeled with the necrotic core, which consisted of highly conductive fluid. When the electric properties of the necrotic core were replaced with poorly conductive tissue, such as white matter, the electric field coverage and rate of energy deposition were increased as shown in the EVH (B) and SARVH (D), respectively. The electric field diagrams showed differences in the electric field coverage at the GTV with (E) and without (F) the necrotic core. EVH, electric field–volume histogram; SARVH, specific absorption rate–volume histogram.

To further investigate the electric field coverage of GTV as influenced by the conductivity of the necrotic core, both EVH and SARVH were constructed as the GTV was modeled with or without the necrotic core. When highly conductive fluid in the necrotic core was replaced with electric conductivity and relative permittivity of white matter, the V_E150_ of the GTV shifted from 20% to 30% (Figs 6A and B) and the corresponding V_SAR7.5_ increased from 13% to 15% (Figs 6C and D), indicating that electric field and SAR coverage of the GTV increased as the highly conductive fluid within the necrotic core is replaced with an electrically lower conductive material property. Because the dielectric properties of GTV and necrotic core probably vary among individual patients, the findings here indicate that acquiring individualized electric conductivity and permittivity values for these structures may have clinical relevance when modeling TTFields in glioblastoma patients.

### The influence of cerebrospinal fluid on GTV

**Figure 7:**
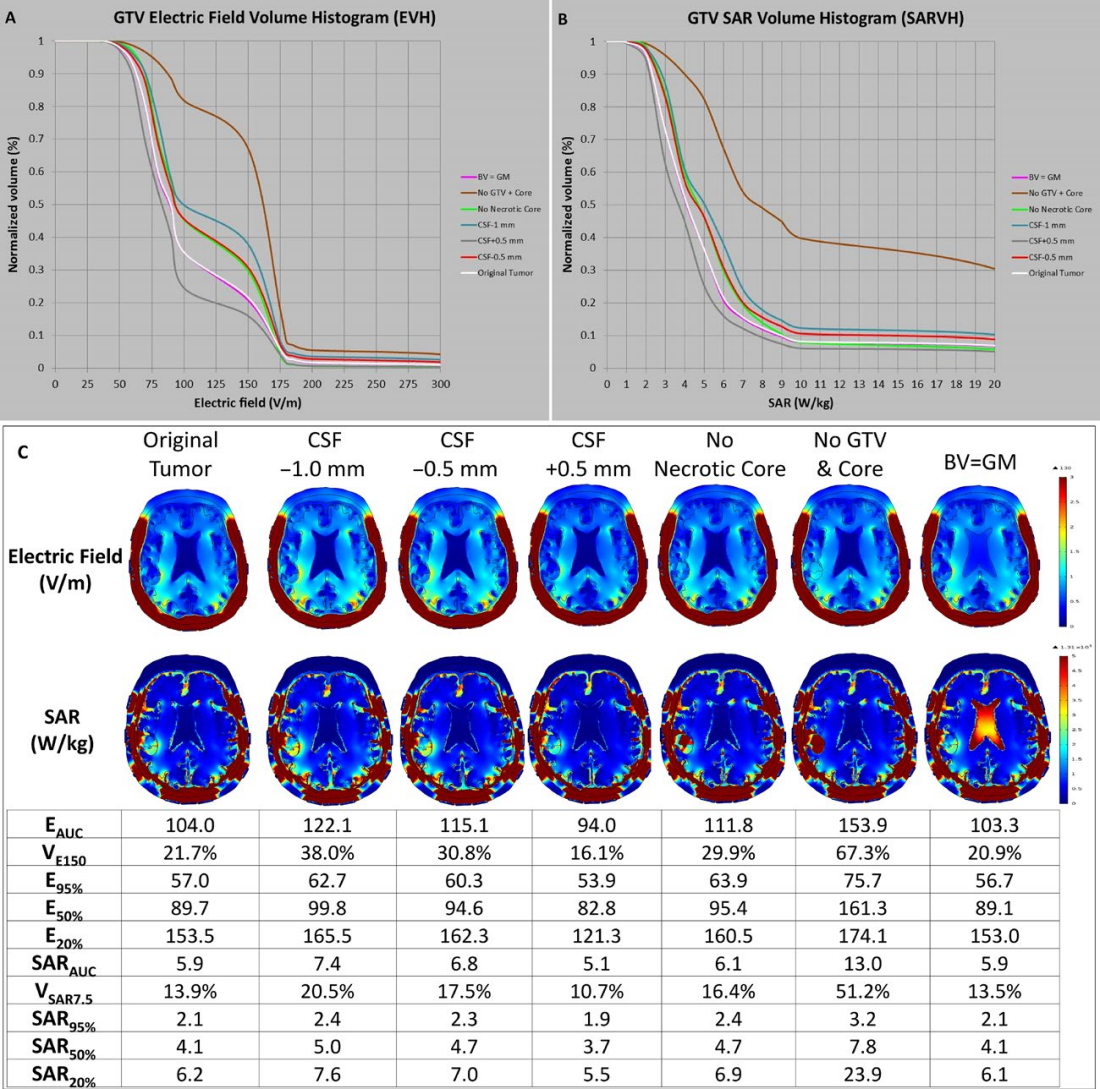
Influence of cerebrospinal fluid on the electric field strength and rate of energy deposition at the GTV and necrotic core. The layer of cerebrospinal fluid was altered by −1.0 mm, −0.5 mm, and +0.5 mm at the convexity of the brain and the respective EVH (A) and SARVH (B) at the GTV were generated. The electric field parameters E_AUC_, V_E150_, and E_50%_, as well as parameters for the rate of energy deposition SAR_AUC_, V_SAR7.5_, and SAR_50%_, all increased progressively when the cerebrospinal fluid space was narrowed progressively from +0.5 mm to −0.5 mm, and then to −1.0 mm on the convexity of the brain. GTV, gross tumor volume; EVH, electric field–volume histogram; SARVH, specific absorption rate–volume histogram; E_AUC_, electric field area under the curve; V_E150_, volume covered with electric field intensity of 150 volts per meter; and E_50%_, the electric field intensity encompassing 50% of volume; SAR, specific absorption rate; SAR_AUC_, SAR area under the curve; V_SAR7.5_, volume covered with specific absorption rate of 7.5 watts per kilogram; SAR_50%_, the magnitude of specific absorption rate encompassing 50% of volume.

As cerebrospinal fluid is a highly conductive medium compared with ordinary brain tissue and it is in close proximity to the GTV, especially in our patient, we sought to investigate whether the cerebrospinal fluid along the surface of the brain and inside the ventricles could influence the electric field and SAR coverage of the GTV. First, 0.5 mm of cerebrospinal fluid was added on the surface or 0.5 mm to 1.0 mm of cerebrospinal fluid was subtracted from the surface of the original brain, corresponding to a respective volume of 275 cc, 196 cc and 179 cc compared to the initial volume of 240 cc. Indeed, E_AUC_, VE_150_, and E_50%_ of the GTV all increased progressively when the cerebrospinal fluid space was narrowed progressively from +0.5 mm to −1.0 mm on the convexities of the brain. Specifically, E_AUC_ increased from 94.0 V/m at +0.5 mm to 115.1 V/m at −0.5 mm, and to 122.1 V/m at −1.0 mm (Fig. 7C). Similarly, the rate of energy deposited in the GTV increased progressively, as represented by SAR_AUC_, V_SAR7.5_, and SAR_50%_, when the cerebrospinal fluid space was narrowed from +0.5 to −1.0 mm on the convexities of the brain. In particular, SAR_AUC_ increased from 5.1 W/kg at +0.5 mm, to 6.8 W/kg at −0.5 mm, and to 7.4 W/kg at −1.0 mm (Fig. 7C). Therefore, increased cerebrospinal fluid space at the convexity shunts electric field and energy away from the brain, whereas decreased cerebrospinal fluid space allows a higher intensity of electric field and SAR to penetrate the brain.

### The influence of the material properties of tumor, necrotic core, and bilateral ventricles on EVH and SARVH

By replacing the material properties of the segmented volume representing the original tumor (GTV + necrotic core) with that of white matter, the electric field strength in terms of E_AUC_, V_E150_, E_95%_, E_50%_, and E_20%_ were all remarkably higher compared with the model containing the original tumor (Fig. 7C); proportionally the SAR was also increased dramatically. The E_AUC_ for the original tumor was 104 V/m in comparison to 153.9 V/m after the change, which is an increase of about 48%. The SAR_AUC_ also increased by 120%, from 5.9 to 13.0 W/kg. Similarly, but not as dramatically, replacing the material properties of the segmented necrotic core with that of white matter increased the electric field strength and SAR within the GTV. Specifically, the E_AUC_ of the replaced necrotic core and the original tumor was 111.8 and 104.0 V/m, respectively. In addition, the SAR_AUC_ of the replaced necrotic core and the original tumor was 6.1 and 5.9 W/kg, respectively. Interestingly, when the material properties of the bilateral ventricle, which was considered a highly conductive medium, were replaced with the properties of gray matter, neither the electric field strength nor SAR changed significantly. This is possibly due to the bulk of the incident electric fields, which permeate first through cerebrospinal fluid at the convexity of the brain, has already deposited most of the energy at the convexity interface as the cerebrospinal fluid in this region is highly conductive compared with adjacent tissues. In contrast, the intensity of the source fields was not easily altered in regions deep within the center of the brain where the bilateral ventricles are located.

### The influence of tumor geometry on EVH and SARVH

**Figure 8:**
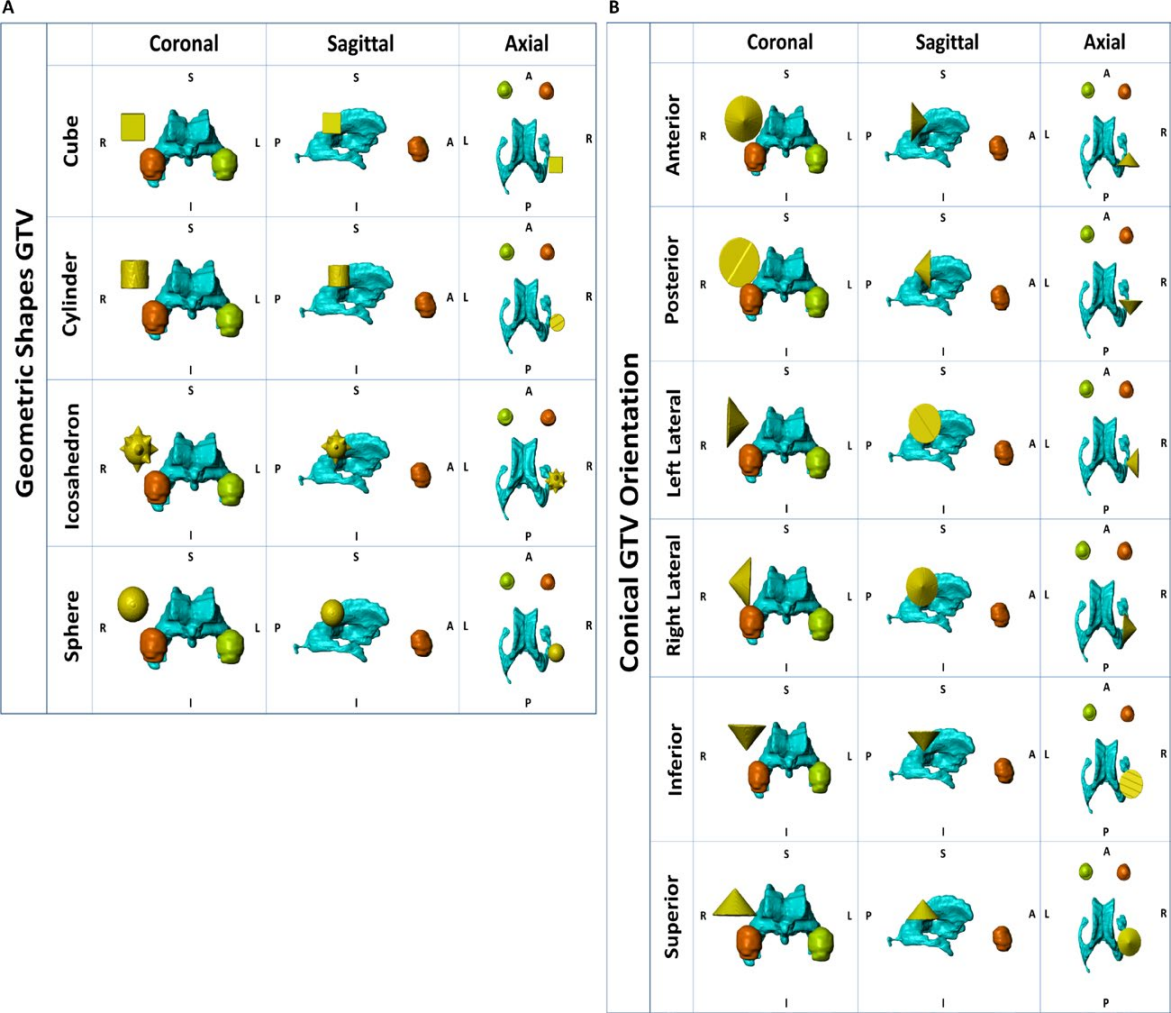
Geometric analysis of GTV. The glioblastoma was represented by standard and relatively symmetric geometric solids, including cube, cylinder, sphere, and icosahedron, for studying changes in electric fields distribution and energy deposition in the GTV (A). The conical shape was also chosen because it is extremely asymmetrical. Its position in the brain, as represented by the anterior, posterior, left lateral, right lateral, inferior, and superior orientations, was also used for studying changes in electric fields distribution and energy deposition in the GTV (B). GTV, gross tumor volume; EVH, electric field–volume histogram; SARVH, specific absorption rate–volume histogram.

The influence of tumor geometry on the electric field and rate of energy deposition was also investigated. The initial GTV was kept intact as segmented in the patient's model, and was used to compare with GTVs of other standard geometric solids, while keeping the centroid of each GTV at the same location. All other parameters such as array position and electric conductivity of tissue remained the same (Fig. 3E and Table 1). Standard geometric solids, including cube, cylinder, sphere, icosahedron, and cone, were used to represent the shape of the tumor for studying changes in electric field distribution and energy deposition in the GTV (Fig. 8). To simplify the comparisons, the necrotic core properties across all models, including the original brain model, were set equal to the GTV; thus, the GTV studied in this section is essentially GTV and necrotic core combined into one entity. In addition, we were interested to determine how the electric fields are distributed depending on the orientation of the conical solid. Therefore, the conical solid was rotated about the geometric centroid of the original patient's GTV, with the tip of the solid pointing in six different directions according to the patient's anterior, posterior, left, right, superior, or inferior head positions.

**Figure 9:**
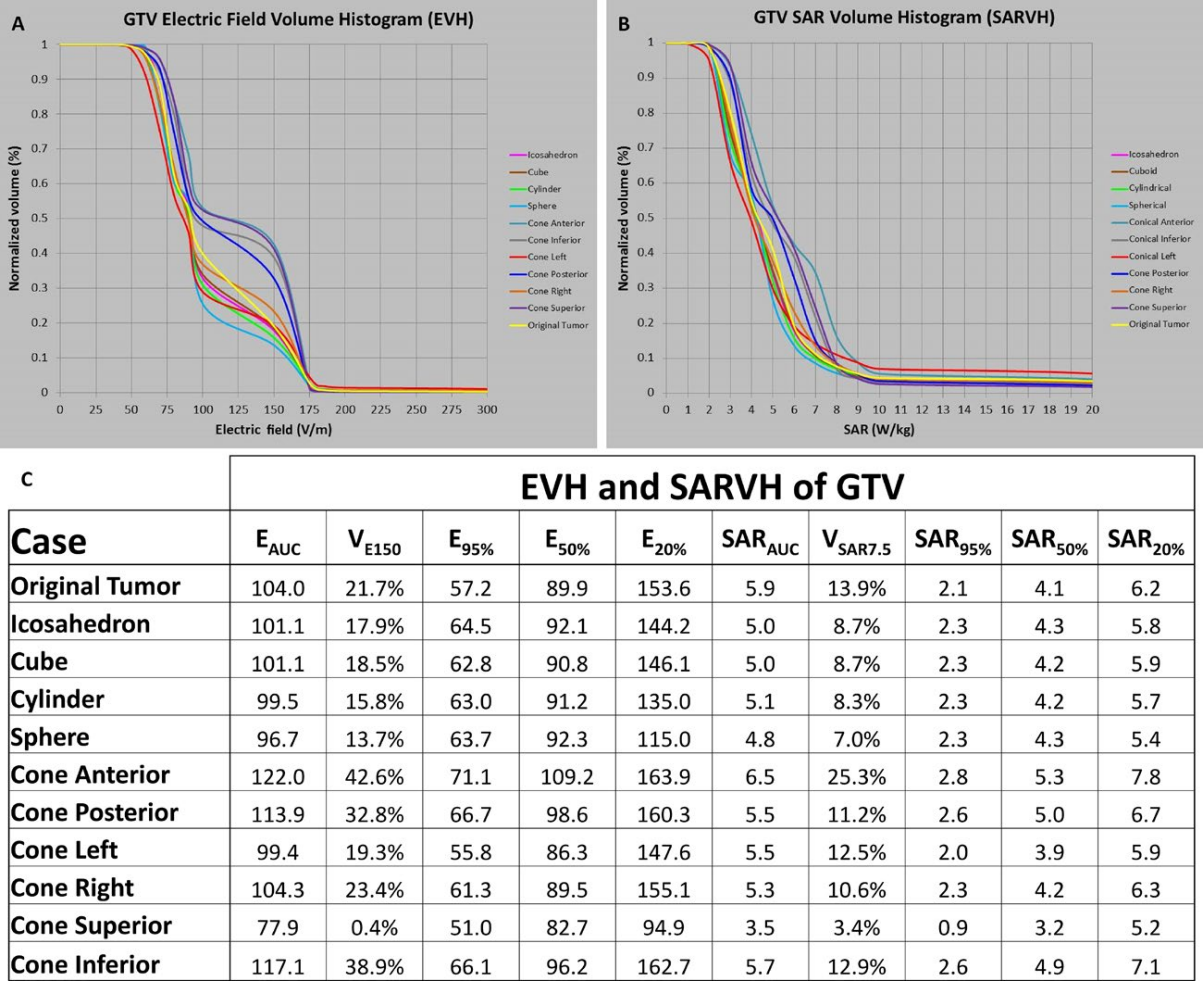
Electric field and rate of energy deposition results in the GTV geometric analysis. EVH was constructed according to the geometries displayed in Figure 8A and B (A). SARVH was constructed according to the geometries displayed in Figures 8A and B. Electric field and SAR results are listed for various GTV geometries (C). GTV, gross tumor volume; EVH, electric field–volume histogram; SARVH, specific absorption rate–volume histogram; E_AUC_, electric field area under the curve; V_E150_, volume covered with electric field intensity of 150 volts per meter; E_95%_, the electric field intensity encompassing 95% of volume; E_50%_, the electric field intensity encompassing 50% of volume; E_20%_, the electric field intensity encompassing 20% of volume; SAR, specific absorption rate; SAR_AUC_, SAR area under the curve; V_SAR7.5_, volume covered with specific absorption rate of 7.5 watts per kilogram; SAR_95%_, the magnitude of specific absorption rate encompassing 95% of volume; SAR_50%_, the magnitude of specific absorption rate encompassing 50% of volume; SAR_20%_, the magnitude of specific absorption rate encompassing 20% of volume.

Compared to the original tumor geometry, symmetric, less angulated geometries had a tendency to associate with a lower basal level of electric fields, as shown by lower values in E_AUC_ and E_20%_, and diminished energy absorption, as shown by SAR_AUC_ and SAR_20%_ (Fig. 9). In particular, the cylindrical and spherical GTVs had lower E_AUC_ and E_20%_, as well as SAR_AUC_ and SAR_20%_, than that for the cube and icosahedron, and these changes are likely due to the fact that the latter ones had more angulated corners. For lesser symmetric geometric shapes, such as a cone, the orientation in three‐dimensional space of the brain was important. The E_AUC_ and E_20%_, as well as SAR_AUC_ and SAR_20%_, had the lowest values when the cone was pointing superiorly but highest when pointing anteriorly (Figs. 8B and  9C). The highest values of E_AUC_ 122.0 V/m and E_20%_ 163.9 V/m, as well as SAR_AUC_ 6.5 W/kg and SAR_20%_ 7.8 W/kg, were found when the conical GTV was pointing anteriorly, with the flat surface facing orthogonal to the lateral ventricle.

## Discussion

As it is being used to treat glioblastoma, TTFields at 200 kHz permeate the patient brain according to the laws of physics, including Gauss’ law, Coulomb's law, and the principles of continuity and capacitance. Our modeling of these fields using the finite‐element method reveals that the intensity of TTFields is highest at the sulci, genu of the corpus callosum, and at the medial surface of the GTV facing the lateral ventricle. To better assess quantitatively the electric field distribution and rate of energy deposition within the brain, volume histograms were constructed for the electric fields and SAR. To our knowledge, this is the first comprehensive usage of both EVH and SARVH to quantify and compare, respectively, TTFields distribution and rate of energy deposition within the patient brain and tumor in various models. Both EVH and SARVH can also facilitate the quantitative comparison of intracranial TTFields between patients, using prespecified values at the GTV and other segmented structures, such as E_AUC_, V_E150_, E_95%_, E_50%_, and E_20%_ for TTFields distribution as well as SAR_AUC_, V_SAR7.5_, SAR_95%_, SAR_50%_, and SAR_20%_ for rate of energy deposition. E_AUC_ represents the aggregate electric fields as represented by the area under the curve within any segmented structures and V_E150_ is the volume of distribution for the electric field intensity at least 150 V/m on the histogram. Similarly, SAR_AUC_ also represents the aggregate rate of energy deposited as represented by the area under the curve within any segmented structures and V_SAR7.5_ is the volume of distribution for SAR at 7.5 W/kg on the histogram. In addition, E_95%_, E_50%_, and E_20%_ are data points on the EVH curve that represent the electric field intensity that covers 95%, 50%, and 20% of the GTV, whereas SAR_95%_, SAR_50%_, and SAR_20%_ are data points on the SARVH that represent the rate of energy deposited in 95%, 50%, and 20% of the GTV. Collectively, these parameters quantitatively represent the intrinsic characteristics of EVH and SARVH and therefore allow the comparison of TTFields among different patients.

Our TTFields modeling is based on MP‐RAGE, T1, and T2 MRI sequences obtained from a patient with glioblastoma in the right parietal brain. Using patient‐based MRI for finite‐element modeling has several distinct advantages compared to modeling based on atlas‐based human head and/or generalized tumor model 10, 11. First, there are intrinsic factors that are unique to the individual human head that may influence the electric field and rate of energy deposition in the GTV. A prime example is the volume of the cerebrospinal fluid within the subarachnoid space at the convexity. Because cerebrospinal fluid is a good electric conductor, a thicker layer tends to shunt TTFields away from the brain while a thinner layer allows more fields to penetrate the brain and eventually to the GTV. Indeed, as brain atrophy occurs in the general population spanning the entire age continuum in adulthood, there is a simultaneous increase in cerebrospinal fluid space from age 18 to 80 12. Furthermore, the rate of atrophy is quicker for men than woman, particularly in the left hemisphere, suggesting neuroanatomical differences between genders 12. Second, the presence or absence of the necrosis within the glioblastoma also can influence the distribution of TTFields at the GTV 12. The necrotic core is primarily consisted of a collection of thick fluid from broken‐down cellular debris containing various proteins and metabolites 13, 14. Because this fluid is ionic and highly conductive, it may direct electric fields toward the more cellular portion of the GTV. Indeed, our modeling has shown that the necrotic core can influence both the electric field strength and SAR within the adjacent GTV as the electric conductivity of GTV varied from 100 to 1 S/m. Lastly, glioblastoma is an infiltrative tumor 15 and the accumulation of the tumor cells in the adjacent gyri and sulci may change the geometry of the tumor. Our geometric analysis has shown that electric field and SAR values grossly increase as the tumor geometry becomes more angulated, as seen in the cube and icosahedron models compared to spherical and cylindrical models. This is likely due to the fundamental basis of electric force and surface charge distribution, where charges bunched up at sharp corners experience a very high internal electric force exerted upon adjacent charges, thereby creating a higher electric field. Likewise, smoother surfaces enable electric charges to distribute more uniformly, generating a lower electric field. An extreme form of tumor geometric asymmetry is represented by a cone and modeling revealed that the orientation of this structure is important, resulting in highest electric field intensity and SAR in this particular patient model when the vertex is pointing anteriorly and the flat base is facing orthogonally to the lateral ventricle. Taken together, atlas‐based modeling may not accurately incorporate patient‐related differences in brain volume, CSF space and physical characteristics of the tumor, all of which influence TTFields distribution in the brains of glioblastoma patients.

Transducer array positioning also influences TTFields distribution within the brain. In accordance to the data present by Wenger et al. 6, we also noted changes in TTFields at the GTV depending on array positioning, with significant variability in the electric field strength of GTV between 100 and 150 V/m, ranging from only 20% volume having 100 V/m with clockwise rotation of the lateral arrays and no displacement of the anterior–posterior arrays to >40% volume having at least 150 V/m when the lateral arrays are rotated in a counterclockwise fashion and the anterior array is moved backward. SAR variability in the rate of energy deposition was less dramatic, as the magnitude of SAR_50%_ changes between 3 and 6 W/kg. These results suggest that array positioning is important to maximize TTFields distribution at the GTV and to determine if the maximal electric field distribution correlates with survival in the patient population treated with TTFields.

Our results have limitations. First, our modeling only used isotropic values but there are local fields generated by the electrical activities of neurons and the flow of cerebrospinal fluid that influence regional electric conductivity 16, 17. Therefore, anisotropic mapping may delineate a more accurate distribution of TTFields. However, for comparing various structural components that may influence TTFields, isotropic modeling still allows a relatively accurate comparison among different dielectric‐, geometric‐, and tumor‐related parameters. Second, the GTV is delineated by the leakage of gadolinium across permeable tumor vasculature. But this vascular permeability can be increased or decreased by radiation and drugs, as in, respectively, pseudoprogression after concurrent radiation and temozolomide treatment for newly diagnosed glioblastoma or pseudoresponse after the administration of an antiangiogenic agent like bevacizumab 18. Therefore, the GTV can be overestimated or underestimated depending on the biological response of the glioblastoma to treatment, and the resolution of this issue is limited by the currently available MRI technology. However, methionine positron emission tomography can help to confirm the presence of active tumor within the gadolinium‐enhanced GTV, whereas diffusion‐weighted MRI and multivoxel MR spectroscopy may detect nonenhancing tumor without hyperpermeable vasculature 19, 20. Third, we did not account for infiltrative nonenhanced disease in the model because the purpose of this study is to address bulk changes in distribution of the TTFields and energy deposition by varying the physical aspects of the treatment delivery. Lastly, we only performed modeling on one patient. It will require comparison of multiple patients, in particular between responders and nonresponders, in order to determine the relevant parameters that influence TTFields in the glioblastoma population.

In conclusion, we constructed volume histograms EVH and SARVH to facilitate future comparisons between glioblastoma patients undergoing treatment with TTFields. TTFields at the GTV are influenced by the dielectric characteristics of the adjacent tissues as well as the GTV itself, particularly the presence or absence of a conductive necrotic core. The amount of cerebrospinal fluid at the convexity of the brain and the geometry of the tumor are additional relevant factors. Finally, the position of the arrays also can influence the electric field distribution and rate of energy deposition within the GTV.

## Conflict of Interest

None declared.

## References

[1] Kirson, E. D. , V. Dbaly , F. Tovarys , J. Vymazal , J. F. Soustiel , A. Itzhaki , et al. 2007 Alternating electric fields arrest cell proliferation in animal tumor models and human brain tumors. Proc. Natl Acad. Sci. USA 104:10152–10157. 17551011 10.1073/pnas.0702916104 PMC1886002

[2] Kirson, E. D. , Z. Gurvich , R. Schneiderman , E. Dekel , A. Itzhaki , Y. Wasserman , et al. 2004 Disruption of cancer cell replication by alternating electric fields. Can. Res. 64:3288–3295. 10.1158/0008-5472.can-04-0083 15126372

[3] Gera, N. , A. Yang , T. S. Holtzman , S. X. Lee , E. T. Wong , and K. D. Swanson . 2015 Tumor treating fields perturb the localization of septins and cause aberrant mitotic exit. PLoS ONE 10:e0125269. 26010837 10.1371/journal.pone.0125269 PMC4444126

[4] Stupp, R. , E. T. Wong , A. A. Kanner , D. Steinberg , H. Engelhard , V. Heidecke , et al. 2012 NovoTTF‐100A versus physician's choice chemotherapy in recurrent glioblastoma: a randomised phase III trial of a novel treatment modality. Eur. J. Cancer 48:2192–2202. 22608262 10.1016/j.ejca.2012.04.011

[5] Stupp, R. , S. Taillibert , A. A. Kanner , S. Kesari , D. M. Steinberg , S. A. Toms , et al. 2015 Maintenance therapy with tumor‐treating fields plus temozolomide vs temozolomide alone for glioblastoma: a randomized clinical trial. JAMA 314:2535–2543. 26670971 10.1001/jama.2015.16669

[6] Wenger, C. , R. Salvador , P. J. Basser , and P. C. Miranda . 2016 Improving tumor treating fields treatment efficacy in patients with glioblastoma using personalized array layouts. Int. J. Radiat. Oncol. Biol. Phys. 94:1137–1143. 26883559 10.1016/j.ijrobp.2015.11.042

[7] Lok, E. , K. D. Swanson , and E. T. Wong . 2015 Tumor treating fields therapy device for glioblastoma: physics and clinical practice considerations. Expert Rev. Med. Devices 12:717–726. 26513694 10.1586/17434440.2015.1086641

[8] Http://Www.Fda.Gov/Ucm/Groups/Fdagov-Public/@Fdagov-Afda-Adcom/Documents/Document/Ucm247168.Pdf.

[9] Www.Itis.Ethzch/Database.

[10] Miranda, P. C. , A. Mekonnen , R. Salvador , and P. J. Basser . 2014 Predicting the electric field distribution in the brain for the treatment of glioblastoma. Phys. Med. Biol. 59:4137–4147. 25003941 10.1088/0031-9155/59/15/4137 PMC4137229

[11] Wenger, C. , R. Salvador , P. J. Basser , and P. C. Miranda . 2015 The electric field distribution in the brain during TTFields therapy and its dependence on tissue dielectric properties and anatomy: a computational study. Phys. Med. Biol. 60:7339–7357. 26350296 10.1088/0031-9155/60/18/7339 PMC4628548

[12] Gur, R. C. , P. D. Mozley , S. M. Resnick , G. L. Gottlieb , M. Kohn , R. Zimmerman , et al. 1991 Gender differences in age effect on brain atrophy measured by magnetic resonance imaging. Proc. Natl Acad. Sci. USA 88:2845–2849. 2011592 10.1073/pnas.88.7.2845 PMC51336

[13] Hoelscher, M. , N. Richter , C. Melle , F. Von Eggeling , A. Schaenzer , and U. Nestler . 2013 SELDI‐TOF analysis of glioblastoma cyst fluid is an approach for assessing cellular protein expression. Neurol. Res. 35:993–1001. 24225180 10.1179/016164113X13756993777580 PMC3823932

[14] Lohle, P. N. , H. A. Wurzer , P. J. Seelen , L. M. Kingma , and K. G. Go . 1998 Analysis of fluid in cysts accompanying various primary and metastatic brain tumours: proteins, lactate and pH. Acta Neurochir. 140:14–19. 9522902 10.1007/s007010050051

[15] Wong, E. T. 2006 Tumor growth, invasion, and angiogenesis in malignant gliomas. J. Neurooncol. 77:295–296. 16314946 10.1007/s11060-005-9042-8

[16] Miranda, P. C. , M. Hallett , and P. J. Basser . 2003 The electric field induced in the brain by magnetic stimulation: a 3‐D finite‐element analysis of the effect of tissue heterogeneity and anisotropy. IEEE Trans. Bio‐Med Eng. 50:1074–1085. 10.1109/TBME.2003.816079 12943275

[17] Wolters, C. H. , A. Anwander , X. Tricoche , D. Weinstein , M. A. Koch , and R. S. Macleod . 2006 Influence of tissue conductivity anisotropy on EEG/MEG field and return current computation in a realistic head model: a simulation and visualization study using high‐resolution finite element modeling. NeuroImage 30:813–826. 16364662 10.1016/j.neuroimage.2005.10.014

[18] Hygino Da Cruz, L. C. Jr , I. Rodriguez , R. C. Domingues , E. L. Gasparetto , and A. G. Sorensen . 2011 Pseudoprogression and pseudoresponse: imaging challenges in the assessment of posttreatment glioma. AJNR Am. J. Neuroradiol. 32:1978–1985. 21393407 10.3174/ajnr.A2397 PMC7964401

[19] Pirzkall, A. , T. R. McKnight , E. E. Graves , et al. 2001 MR‐spectroscopy guided target delineation for high‐grade gliomas. Int. J. Radiat. Oncol. Biol. Phys. 50:915–920. 11429219 10.1016/s0360-3016(01)01548-6

[20] Dhermain, F. G. , P. Hau , H. Lanfermann , A. H. Jacobs , and M. J. van den Bent . 2010 Advanced MRI and PET for assessment of treatment response in patients with gliomas. Lancet Neurol. 9:906–920. 20705518 10.1016/S1474-4422(10)70181-2

